# The effects of pre-existing dementia on surgical outcomes in emergent and nonemergent general surgical procedures: assessing differences in surgical risk with dementia

**DOI:** 10.1186/s12877-018-0844-x

**Published:** 2018-07-03

**Authors:** Woubet Tefera Kassahun

**Affiliations:** Clinic for Visceral, Transplantation, Thoracic and Vascular Surgery, University of Leipzig, Liebig Strasse 20, 04103 Leipzig, Germany

**Keywords:** Dementia, Surgical outcomes, Morbidity, Mortality, Predictive factors

## Abstract

**Background:**

The aim was to assess the morbidity and in-hospital mortality that occur in surgical patients with pre-existing dementia compared with those outcomes in non-dementia patients following emergent and nonemergent general surgical operations.

**Methods:**

A total of 120 patients with dementia were matched for sex and type of surgery with 120 patients who did not have dementia, taken from a cohort of 15,295 patients undergoing surgery, in order to assess differences in surgical risk with dementia. Patient information was examined, including sex, body mass index (BMI), prevalence of individual comorbidities at admission, and several other variables that may be associated with postoperative outcomes as potential confounders.

**Results:**

Patients with dementia tended to have a higher overall complication burden compared to those without. This was evidenced by a higher average number of complications per patient (3.30 vs 2.36) and a higher average score on the comprehensive complication index (48.61 vs 37.60), values that were statistically significant for a difference between the two groups. The overall in-hospital mortality in patients with dementia was 28.3% (34 deaths out of 120 patients). During the same period, at our hospital, the overall in-hospital mortality in the control group was 20% (24 deaths out of 120 patients). Patient groups with and without dementia each had 3 and 5 associated risk factors for morbidity and 9 and 12 risk factors for mortality, respectively.

**Conclusions:**

Patients with pre-existing dementia have a greater than average risk of early death after surgery, and their incidence of fatal complications is higher than that of surgical patients without dementia.

## Background

Dementia represents a chronic global loss of cognitive or brain function and manifests as the loss of memory, executive function and attention [1, 2]. Although dementia can affect a person at any age, those at most risk are essentially older people. Worldwide, the population aged 80 and older is expected to increase from 126.5 million in 2015 to 446.6 million in 2050 [3].This means that as older age groups increase in size, the global prevalence of dementia in the world population will substantially increase, with estimates suggesting 65.7 million by 2030 and a near doubling to 115.4 million by 2050 [2, 4]. Given these demographic changes, a rise in the potential number of surgical patients with dementia can also be expected. Thus, the demand for the care and treatment of the older patients with dementia and surgical problems is likely to grow in the next years.

Surgical procedures in patients with dementia carry a significant risk of complications and have a high mortality rate. In one recent study [5], surgical mortality for the patient with dementia was 13% in 30 days, increasing with time to as high as 92% in two years, compared with a surgical mortality rate of less than 7% for those without dementia [6, 7]. As the mortality rates for many leading causes of death have declined over the past decade, these high mortality rates for dementia have not improved significantly and may increase further.

Moreover, with a projected survival of 3–12 years from diagnosis, these patients have a shorter life expectancy than those without dementia [8–10]. Accurate preoperative risk stratification can be difficult because pre-existing dementia that contributes to the early death of such patients is a non-modifiable factor. Thus, the treatment of choice for this group of patients is difficult to determine.

Previous studies dealing with surgical outcomes among patients with pre-existing dementia have concentrated mainly on traumatic patients [11–13]. There have been few studies in patient populations with general and vascular surgical conditions and dementia [5, 6, 14], and their findings have not been consistent. There has not been a study, to our knowledge, that compared outcome after surgery among non-traumatic patients with a pre-existing diagnosis of dementia with outcomes among an equal number of operated patients who did not have dementia, matched for sex, type of surgery and with relatively similar patient characteristics and surgical variables. Understanding clinical conditions unique to older adults that affect surgical outcomes is important. Dementia for any reason is currently not part of any routinely performed pre-surgical assessment strategy in general surgery. As a result, little is known about the effects of pre-existing dementia on postoperative outcomes.

This study was done retrospectively. Data have been generated to identify patient-, disease-, and management-related factors that were associated with adverse outcomes in these patients. Its purpose was to evaluate surgical outcomes among non-traumatic patients with pre-existing dementia and to compare these outcomes with those of sex- and treatment-matched controls without dementia in an attempt to identify predictors of morbidity and early death.

## Methods

Data from a database of the Department of Transplantation, Thoracic, Visceral and Vascular Surgery of the University of Leipzig were retrospectively analyzed for the years 2011 to 2017. This included review and analysis of data for all studied patients who had been prospectively entered in a data registry, which records patient and disease characteristics and outcomes. Based on the principal operative procedure, all elective or emergent operations were categorized as involving general surgery (GS) and vascular surgery (VS).

Only patients whose procedure warranted more than an overnight stay were selected. All patients with pre-existing dementia (*n* = 120) who underwent surgery between November 2011 and August 2017 at our center were included in this study. Dementia was defined as any outpatient physician visits or hospital admissions in which dementia was recorded as a diagnosis according to the International Statistical Classification of Diseases and Related Health Problems, tenth edition [ICD-10; F00, F01, F02, F03 or G30]. Patients with mental status changes or delirium in the context of their current illness were not included in this study.

**Fig. 1:**
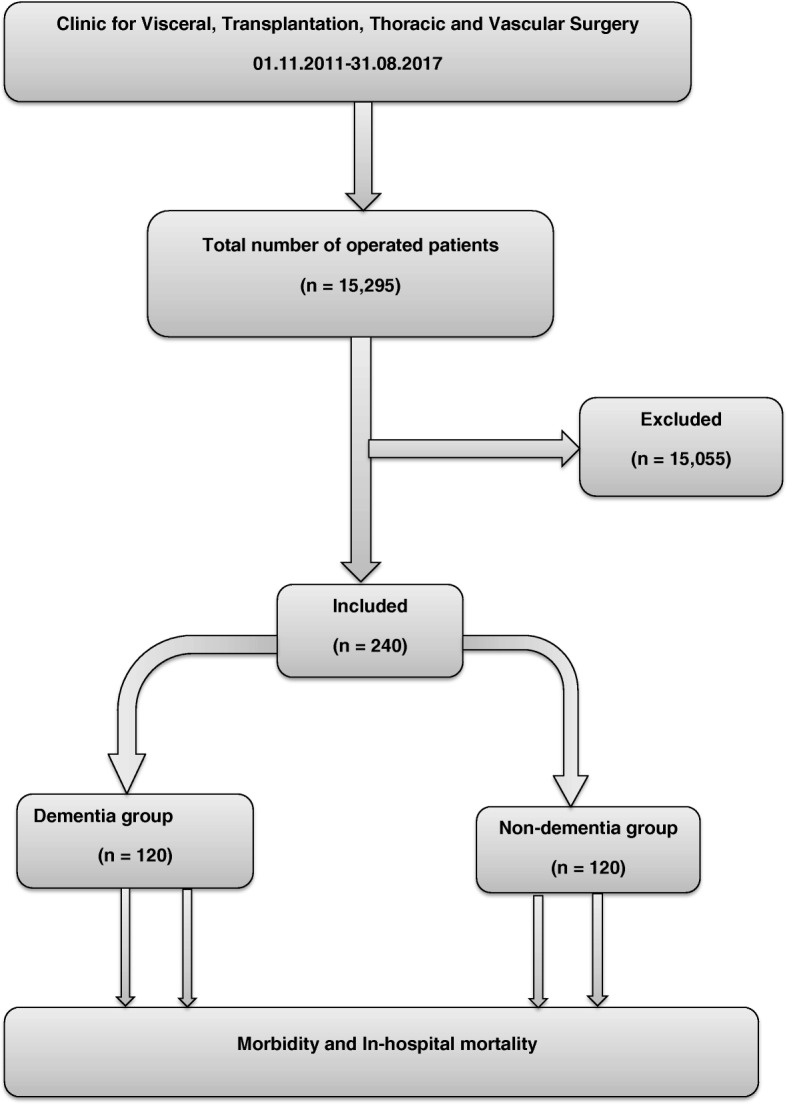
Flow diagram of patient selection

In order to evaluate differences in surgical risk associated with dementia, the 120 patients with dementia were matched for sex and type of surgery with equal number of controls who did not have dementia taken from a cohort of 15,295 surgical patients (Fig. 1). Patient Characteristics (Table 1) and surgical variables (Table 2) that may be associated with postoperative outcomes as potential confounders were examined. In cases of multiple procedures on a patient during hospitalization, only the initial procedure was eligible for inclusion. The main outcome measures were morbidity and in-hospital mortality (End of follow-up was discharge from the hospital, and mortality was defined as hospital death). The severity of medical conditions at the time of surgery was evaluated using the American Society of Anesthesiologists (ASA) Physical Status classification [15]. The Clavien-Dindo classification (CDC) of surgical complications [16] was used to classify surgical complications. In addition, based on CDC at discharge, the comprehensive complication index (CCI) [17] was calculated for each patient in order to evaluate the true overall morbidity burden of a procedure.

**Table 1:** Patient characteristics by group

Variable	Dementia group	Non-dementia group	*p*-value
	(*n* = 120)	(*n* = 120)	
Sex			
Female	71 (59.2)	71 (59.2)	1.00
Male	49 (40.8)	49 (40.8)	1.00
Age, years, mean ± SD	80.45 ± 9.07	74.06 ± 9.74	.045
BMI, mean ± SD	25.28 ± 5.17	26.78 ± 6.60	.055
COD			
Hypertension	109 (90.8)	100 (83.3)	.083
Congestive heart failure	46 (38.3)	32 (26.7)	.054
Ischemic heart disease	27 (22.5)	32 (26.7)	.454
Cardiac arrhythmia	58 (48.3)	39 (32.5)	.012
Cardiac valve disease	16 (13.3)	11 (9.2)	.307
Diabetes mellitus	47 (39.2)	47 (39.2)	1.00
COPD	20 (16.7)	14 (11.7)	.267
Chronic renal failure	44 (36.7)	29 (24.2)	.035
Vascular disease	56 (46.7)	44 (36.7)	.116
CNS disease	38 (31.7)	18 (15.0)	.002
COD-PP, mean ± SD	4.99 ± 2.39	4.49 ± 2.51	.116
ASA-Class^χ^			
ASA 1	0 (0.0)	1 (0.8)	.333
ASA 2	11 (9.2)	23 (19.2)	.044
ASA 3	70 (58.3)	70 (58.3)	1.00
ASA 4	23 (19.2)	22 (18.3)	.666
ASA 5	5 (4.2)	1 (0.8)	.081
PS-PP, mean ± SD	1.33 ± 1.76	1.68 ± 1.94	.144
Disease entity			
Benign	102 (85)	103 (85.8)	.711
Malignant	18 (15)	17 (14.2)	.711

*n* total number of patients, *SD* standard deviation, *BMI* body mass index, *COD* Coexisting disease, *PP* per patient, *COPD* chronic obstructive lung disease, *ASA* The American Society of Anesthesiologists Physical Status classification, *CNS* indicates central nervous system disease and holds for patients with medically documented cerebral vascular accident, transient ischemic attack, or neurological deficit of central origin, *PS* previous surgery; Numbers in bracket show values presented in n (%) unless noted otherwise. χ, Percents may not total 100 due to missing data

**Table 2:** Surgical variables by group

Variable	Dementia group	Non-dementia group	*p*-value
	(*n* = 120)	(*n* = 120)	
Types of surgery			
GS	92 (76.7)	92 (76.7)	1.00
VS	28 (23.3)	28 (23.3)	1.00
Surgical indications			
Critical limb ischemia	23 ((19.2)	19 (15.8)	.732
Bowel obstruction	14 (11.7)	12 (10)	.836
Perforated viscus	13 (10.8)	13 (10.8)	1.00
Decubitus ulcer	9 (7.5)	6 (5.0)	.595
Cholecystitis	8 (6.7)	9 (7.5)	1.00
Cancer GIT	7 (5.8)	7 (5.8)	1.00
Diverticulitis	6 (5.0)	10 (8.3)	.439
Hernia	6 (5.0)	8 (6.7)	.784
Mesenteric ischemia	6 (5.0)	2 (1.7)	.281
Diabetic angiopathy	5 (4.2)	4 (3.3)	1.00
Miscellaneous	23 (19.2)	30 (25.0)	.631
Surgical treatment			
Amputation	16 (13.3)	14 (11.7)	.846
Bowel resection	22 (18.3)	19 (15.8)	.732
Surgical revascularization	16 (13.3)	16 (13.3)	1.00
Adhäsiolysis	7 (5.8)	7 (5.8)	1.00
Major resection HBP	3 (2.5)	3 (2.5)	1.00
Cholecystectomy	8 (6.7)	9 (7.5)	1.00
Thyroidectomy	4 (3.3)	4 (3.3)	1.00
Multivisceral resection	5 (4.2)	4 (3.3)	1.00
Closure perforated viscus	8 (6.7)	10 (8.3)	.807
Hernia repair	7 (5.8)	8 (6.7)	1.00
Procedures thorax	4 (3.3)	4 (3.3)	1.00
Miscellaneous	20 (16.7)	22 (18.3)	.816
Surgical technique			
Conventional	101 (84.2)	103 (85.8)	.718
Minimally invasive	17 (14.2)	16 (13.3)	1.00
Hybrid	2 (1.7)	2 (1.7)	1.00
Urgency			
Emergency	61 (50.8)	57 (47.5)	.606
Elective	59 (49.2)	63 (52.5)	.606
Classification of OT			
OT < 90 min	58 (48.3)	56 (46.7)	.796
OT ≥ 90 min	62 (51.7)	64 (53.3)	.796
OT, mean ± SD	103.78 ± 80.17	119.68 ± 94.93	.162

*n* total number of patients, *GS* general surgery, *VS* vascular surgery, *OT* operative time; Numbers in bracket show values presented in n (%) unless noted otherwise

Statistical analysis was performed using SPSS software version 24 for windows (IBM Corporation, USA). All statistical tests were 2-sided, and a *P* value ≤0.05 was considered statistically significant. Descriptive statistics assessed the distribution of patients, procedures, comorbidities, morbidity and mortality by group. Univariate statistical comparisons between groups were performed using Student’s t-test for continuous variables and the chi-square test for discrete variables to examine the univariate relation between preoperative risk factors and outcome variables. Based on the sample size, those risk factors related to morbidity and mortality at a 0.05 significance level were then entered into a multivariate logistic regression analysis, with outcome variables as dependent variables and the risk factors as independent variables, to identify clinical features that were predictive of morbidity and mortality associated with patient groups. This study was approved by the institutional ethics committee review board of the medical faculty of the University of Leipzig in Leipzig, Germany.

## Results

The current study reports the relationship between pre-existing dementia and postoperative outcomes. A total of 15,295 patients who had surgery in our hospital from November 2011 to August 2017 were identified. Among these, 240 patients were studied. Stratification by diagnosis yielded 120 patients with pre-existing dementia and a female predominance for undergoing elective and emergent operations in general and vascular surgery. These patients were matched for sex and type of surgery with 120 patients who did not have dementia with a relatively similar distribution of patient characteristics and surgical variables. Almost all variables that define preoperative patient characteristics and surgery were well balanced between the dementia and non-dementia groups. Only 4 of 57 variables (Tables 1 and 2) had a significant difference.

Of the 120 patients with dementia, 71 were female (59.5%). Patients with dementia were older on average (80.5 vs 74.1 years old).

Comorbid conditions that were advanced and stable were present in almost all patients with and without dementia. Their distribution was comparable across both patient groups with the exception of cardiac arrhythmia, chronic renal failure, and CNS disorders, which tended to be more frequent among patients with dementia. Otherwise, no significant dementia-related differences in patient characteristics were observed in the study population. Furthermore, as Table 2 shows, the distributions of the type of surgery, surgical indications, specific type of surgical procedures, surgical techniques, urgency, and mean operative time are relatively similar in both groups.

A summary of surgical outcome data is depicted in Table 3. As this table shows, the occurrence of postoperative complications evaluated using the CDC is not as different, as expected, in the patients with dementia compared to those without dementia and is relatively comparable across both patient groups. In addition, based on the CDC at discharge, the CCI was calculated retrospectively, taking into account all complications after a procedure and their respective severity, in an effort to quantitate and compare the true overall morbidity burden of a procedure. In contrast to the CDC, when we evaluate the true overall morbidity burden of a procedure using the CCI, individuals with dementia tended to have a significantly higher score compared with those without. This indicates a higher overall complication burden in this group. This was also evidenced by a higher average number of complications per patient, which was statistically significant for a difference between the two groups. Moreover, among specific complications, there was a statistically significant difference between patients with and without dementia when we considered the incidence of surgical site infection (SSI), postoperative delirium (PD) and pneumonia. The occurrence of these complications was significantly higher in patients with pre-existing dementia compared to those without.

**Table 3:** Surgical outcome data by group

Variable	Dementia group	Non-dementia group	*p*-value
	(*n* = 120)	(*n* = 120)	
Complications			
Bleeding	10 (8.3)	16 (13.3)	.299
SSI	57 (47.5)	39 (32.5)	.018
IMDRB	42 (35)	30 (25)	.091
WDN	11 (9.2)	7 (5.8)	.463
Bilioma-Bile leakage	2 (1.7)	6 (5)	.281
Anastomotic leakage	6 (5)	6 (5)	1.00
Sepsis	21 (17.5)	24 (20)	.864
RLN Palsy	2 (1.7)	0 (0)	.498
Hypocalcemia after TX	4 (3.3)	2 (1.7)	.684
Pneumonia	32 (26.7)	10 (8.3)	<.001
Thromboembolism	11 (9.2)	12 (10)	.826
Myocardial infarction	1 (0.8)	2 (1.7)	.561
ARF	22 (18.3)	23 (19.2)	.869
CA new	9 (7.5)	7 (5.8)	.605
Diarrhea	11 (9.1)	7 (5.8)	.424
TIA, Stroke	3 (12.5)	0 (0)	.156
Postoperative delirium	39 (32.5)	3 (2.5)	<.001
Pancreatitis	2 (1.7)	1 (0.8)	.561
LFRI	28 (23.3)	23 (19.2)	.430
PERI	21 (17.5)	16 (13.3)	.371
Complications pp, mean ± SD	3.30 ± 2.71	2.36 ± 2.49	.005
CDC			
Grade I	6 (5)	10 (8.3)	.301
Grade II	27 (22.5)	20 (16.7)	.255
Grade IIIa	7 (5.8)	7 (5.8)	1.00
Grade IIIb	17 (14.2)	24 (20)	.230
Grade Iva	5 (4.2)	3 (2.5)	.472
Grade IVb	3 (2.5)	0 (0)	.247
Grade V	34 (28.3)	24 (20)	.132
CCI, mean ± SD	48.61 ± 37.85	37.6 ± 36.32	.022
Admission to ICU			
Yes	73 (60.8)	62 (51.7)	.152
Reoperation			
One	18 (15)	21 (17.5)	.600
Multiple	22 (18.3)	20 (16.7)	.497
Outcome			
Discharge	86 (71.7)	96 (80)	.132
Death	34 (28.3)	24 (20)	.132
Cause of death			
MI, cardiogenic shock	0 (0)	1 (4.0)	.414
Sepsis with mof	18 (52.9)	19 (79.2)	.041
Decomp. Cardiac GI	8 (23.5)	1 (4.0)	.045
Malignancy final stage	2 (5.9)	0 (0)	.506
Unclear	6 (17.6)	3 (12.5)	.722
Place of death			
ICU	14 (38.24)	21 (87.50)	<.001
Ward	21 (61.76)	3 (12.50)	<.001
Time to death, days			
1–7	14 (41.2)	6 (30.0)	.290
8–14	5 (14.7)	4 (16.7)	.922
15–40	10 (21.4)	11 (45.8)	.419
41–90	2 (5.9)	3 (12.5)	.679
LOS, days, mean ± SD	21 ± 17.98	20.1 ± 16.93	.690

*RLN* recurrent laryngeal nerve, *TX* thyroidectomy, *WDN* wound dehiscence noninfectious, *mof* multiorgan failure, *CA* cardiac arrhythmias, *ARF* acute renal failure, *TIA* transitory ischemic attack, *LFRI* lung failure requiring intubation, *PERI* pleural effusion requiring drainage, *PP* per patient, *CDC* Clavien-Dindo classification of complications, *CCI* comprehensive complication index, *MDRB* multi-drug-resistant bacteria, *LOS* length of hospital stay, *ICU* intensive care unit, *SSI* surgical site infection defined as being contained within the skin or subcutaneous tissue (superficial), or involving the muscle and/or fascia (deep); Numbers in bracket show values presented in n (%) unless noted otherwise

Furthermore, the presence of dementia was associated with an increased likelihood of being admitted to the ICU. In addition, individuals with dementia were more likely to die within the first 7 days of surgical treatment before leaving the ICU.

For patients with dementia, the overall in-hospital mortality rate was 28.3% (34 of 120). Of these 34 deaths, 23 (67.6%) were associated with emergency operations and 11 (32.6%) with elective operations. In-hospital mortality during the same period was 20% (24 of 120) in the non-dementia group. Of these 24 deaths, 17 (70.8%) were associated with emergency operations and 7 (29.2%) with elective operations. The emergency and elective mortality rates were 37.7 and 18.6% in patients with dementia and 29.8 and 11.1% in the group without dementia, respectively. Thus, the postoperative risk of mortality was more than twofold in patients undergoing emergency operations when compared with those undergoing elective operations.

Overall, morbidity and in-hospital mortality were higher in surgical patients with a pre-existing diagnosis of dementia than in the control group. The associations between risk factors, morbidity and in-hospital mortality calculated with the chi-square test for a linear trend by group are depicted in Tables 4 and 5. We performed multivariate analysis with age, ASA classification, pre-existing cardiac arrhythmia, diabetes mellitus, emergent operations, pulmonary complications and surgical site infection as covariates. In this model, emergent operation, ASA class above 2 and pulmonary complications remained significantly associated with surgical outcome (Table 6).

**Table 4:** Associations between risk factors and occurrence of postoperative complications calculated with chi-square for linear trend

	Dementia group (N, 120)			Non-dementia group (N, 120)		
	Occurrence of complication			Occurrence of complication		
	Yes	No	*p* value	Yes	No	*p* value
	*n* = 100	*n* = 20		*n* = 89	*n* = 31	
Risk factor						
Age ≥ 75 years	81 (81)	11 (55)	.012	47 (52.8)	15 (48.4)	.67
Congestive herart disease	41 (41)	5 (25)	.179	29 (32.6)	3 (9.7)	.013
Cardiac arrhythmia	53 (53)	5 (25)	.022	33 (37.1)	6 (19.4)	.070
Diabetes	40 (40)	7 (35)	.676	41 (46.1)	6 (19.4)	.009
Kidney disease	37 (37)	7 (35)	.865	26 (29.2)	3 (9.7)	.029
ASA classification > 2	82 (91.2)	16 (84.2)	.364	76 (87.4)	17 (56.7)	<.001
Emergent operation	56 (56)	5 (25)	.011	48 (53.9)	9 (29)	.017

Numbers in bracket show values presented in n (%) unless noted otherwise

**Table 5:** Associations between risk factors and in-hospital mortality calculated with chi-square for linear trend

	Dementia group (N, 120)			Non-dementia group (N, 120)		
	Outcome			Outcome		
	Survived	Died		Survived	Died	
	*n* = 86	*n* = 34	*p* value	*n* = 96	*n* = 24	*p* value
Risk factor						
Age ≥ 75 years	61 (70.9))	31 (91.2)	.018	47 (49)	15 (62.5)	.235
Cardiac arrhythmia	39 (45.3)	19 (55.9)	.298	27 (28.1)	12 (50)	.041
ASA classification > 2	68 (87.2)	30 (98.3)	.134	69 (74.2)	24 (100)	.005
Emergent operation	38 (44.2)	23 (67.6)	.021	40 (41.7)	17 (70.8)	.010
Surgical site infection	40 (46.5)	17 (50)	.730	27 (28.1)	12 (50)	.041
Sepsis	3 (3.5)	18 (52.9)	<.001	4 (4.2)	20 (83.3)	<.001
Pneumonia	17 (19.8)	15 (44.1)	.007	4 (4.2)	6 (25)	.001
Lung failure	8 (9.3)	20 (58.8)	<.001	1 (1)	22 (91.7)	<.001
Pleural effusion	13 (15.1)	8 (23.5)	.274	8 (8.3)	8 (33.3)	.001
Pulmonary complication	21 (24.4)	28 (82.4)	<.001	12 (12.5)	21 (87.5)	<.001
Cardiovascular complication	7 (8.1)	10 (29.4)	.003	8 (8.3)	11 (45.8)	<.001
Acute renal failure	4 (4.7)	18 (52.9)	<.001	3 (3.1)	20 (83.3)	<.001
Postoperative delirium	21 (24.4)	18 (52.9)	.003	0 (0)	3 (12.5)	<.001

Numbers in bracket show values presented in n (%) unless noted otherwise; cardiovascular complications indicate the total number of cardiovascular complications and include thromboembolism, myocardial infarction and newly diagnosed cardiac arrhythmias. Pulmonary complications indicate the total number of pulmonary complications and include pneumonia, lung failure requiring intubation and pleural effusion requiring drainage

**Table 6:** Multivariable Logistic Regression Analyses

	Dementia group		Non-dementia group	
Predictive factors	Odds ratio (95% CI)	*p* value	Odds ratio (95% CI)	*p* value
Predictive factors for morbidity by group				
Emergent operations	3.56 (1.0–12.67)	.05	3.20 (1.20–8.55)	.02
ASA classification > 2	1.15 (0.41–3.22)	.79	.38 (.17–.84)	.02
Cardiac arrhythmia	2.87 (0.91–9.02)	.07	2.05 (0.68–6.19)	.21
Diabetes mellitus	.89 (0.30–2.66)	.84	2.77 (0.94–8.21)	.07
Age ≥ 75 years	0.34 (0.11–1.04)	.06	1.50 (.56–3.92)	.41
Predictive factors for mortality by group				
ASA classification > 2	2.98 (1.22–7.26)	.02	5.18 (1.23–21.83)	.03
Pulmonary complication	.07 (.02–.23)	<.001	.02 (.002–.09)	<.001
Wound complication	1.28 (.43–3.84)	.66	.15 (.02–.93)	.04
Age ≥ 75 years	4.04 (.91–17.90)	.07	2.12 (.40–11.15)	.38
Cardiac arrhythmia	.96 (.34–2.67)	.94	1.87 (.36–9.73)	.48
Emergent operation	1.34 (.43–4.54)	.59	0.32 (0.07–1.40)	.13

*ASA* The American Society of Anesthesiologists Physical Status classification

## Discussion

The evaluation of risk factors in predicting outcomes in patients with a diagnosis of pre-existing dementia undergoing a variety of general and vascular surgical procedures was the focus of the current study. The hypothesis was that dementia is a surgical factor distinct from sex, comorbidity, and type of surgery and correlates with morbidity and surgical mortality. To examine this assertion, patients with pre-existing dementia were compared with an equal number of patients without dementia matched for sex and type of surgery. Assuming that the determinants of surgical outcome are multifactorial, we analyzed a number of clinical variables. The main result of this study was that, regardless of the advances made in surgical technique and preoperative and postoperative care, outcomes among dementia patients requiring surgery were relatively poor. Compared to patients who did not have dementia, we observed an increased rate of complications and surgical mortality. Of the 120 consecutive surgical patients with pre-existing dementia treated over a 6-year period, 34 (28.3%) died within 90 days of surgery. Sepsis with multi-organ failure and decompensated cardiac global insufficiency were the most common causes of early death.

Previous studies reporting mortality from different data bases describe early mortality rates of 7–13% for surgical patients with pre-existing dementia [5–7]. The mortality rate in the current cohort was generally higher in comparison; however, it should be noted that 50.8% of our patients with dementia and 47.5% without were operated on in emergency sessions. Emergent operation has been recognized as a common determinant of in-hospital mortality [18]. This was also observed in the current study, in which almost 68% of early deaths in the dementia group and 71% in the non-dementia group were after emergent operations. This suggests advanced disease processes at the time of admission.

Among comorbid conditions, the presence of cardiac arrhythmia, chronic renal failure, and CNS disorders was significantly higher in patients with dementia than in those without. However, none of these clinical conditions predicted in-hospital mortality in this group of patients. In this respect, our study extends prior research showing no direct relationship between mortality and the presence of comorbid conditions [19] and indicates dementia by itself as a terminal illness and main determinant of early death.

Furthermore, dementia is an independent risk factor for the development of multiple postoperative complications, particularly postoperative delirium (PD), which is also a major risk factor for postoperative mortality [20–25]. Recently, Mosk et al. [25] observed PD in 34.2% of dementia patients following hip fracture surgery. In agreement with this, the current study found a significantly increased incidence of PD in patients with pre-existing dementia in comparison with those without. The increased incidence of PD (33%) in patients with pre-existing dementia is not surprising because in vulnerable patients, such as those with pre-existing dementia, even a seemingly minor insult such as minor surgery might be enough to precipitate delirium. Conversely, in younger patients without dementia, delirium may develop only after exposure to a series of noxious insults, such as general anesthesia, major surgery and a stay in the ICU [23]. In full agreement with this, in the current study, with only 3 out of 120 patients in the non-dementia group developing this complication, PD was an extremely rare occurrence in this group of patients.

In addition, the occurrence of postoperative delirium correlated strongly with urgent operations, longer intensive care unit stays and longer overall hospital stays (data not shown), emphasizing the need for early diagnosis and aggressive therapy. This agrees with previous research that found an overall longer hospital stay in dementia patients with delirium [26] and an association with an up to fourfold increase in mortality following surgery [20–22, 24, 25, 27].

Hu et al. [14] found pneumonia to be one of the major complications that occurs frequently in surgical patients with pre-existing dementia compared with those patients without. This agrees with our result that showed a significantly higher incidence of postoperative pneumonia in dementia patients. The pneumonia rate among these patients was three times that among sex- and treatment-matched controls. The mortality rate after the development of pneumonia was substantially higher (41%) than the mortality rate for patients in whom such a complication had not developed after surgery. The inability of dementia patients to communicate reasonably and their related inability to participate fully in aggressive postoperative pulmonary exercises, toileting, and ambulation may explain the increased incidence of postoperative pneumonia. Interestingly, however, contrary to other studies that found COPD as a risk factor for pulmonary complications [28], postoperative pneumonia did not correlate with the presence of COPD as a coexisting disease in the present study. However, due to the relatively low prevalence of COPD in the studied patients, this notion may not reflect accurately the influence of pre-existing COPD on the incidence of postoperative pneumonia.

Surgical site infection, acute renal failure requiring dialysis and lung failure requiring intubation are also common postoperative complications, and survival was poor after the onset of these complications. Thus, the combined higher incidence of these adverse postoperative events could lead to a comparably increased risk of early death. Overall, however, although these complications may be heralds of early death, it is the pre-existing condition, in this case, dementia, that is the major problem and underlying cause of death.

Taken as a whole, while treating surgical patients with pre-existing dementia, surgeons should be aware of the limited life expectancy, poor prognosis and the expected severe and multiple complications. With the exception of emergency situations, the indication of burdensome surgical interventions of questionable benefit should be assessed critically, unless this step is necessary to reduce physical suffering. If available, a conservative treatment approach is a more viable option in this difficult to treat patient population.

Several limitations of this study deserve comment. First, we did not have detailed information on the severity of dementia. Thus, it is possible that some individuals with mild cognitive impairment may not have been identified. Accordingly, the presented results may not represent the outcomes of patients with mild dementia that has not yet been clinically recognized by a physician.

Second, specific surgical procedures in this study are heterogeneous. Included procedures that ranged from adhesiolysis to multi-visceral resection do not provide a uniform baseline surgical stress, which leads to variability in measurements such as operative time, requirement of a postoperative ICU stay and length of hospital stay. However, relatively similar types and numbers of operations were performed in patients with and without dementia.

Third, this study is limited in its ability to draw strong conclusions regarding the outcomes of surgery among patients with coexisting dementia compared to patients without. The descriptive analysis employed suggests differences among the groups for some patient and surgical variables including age.

Finally, we were limited also by the retrospective nature of our study and the short-term follow-up of our patient cohort.

Overall, however, the outcome of an institution-based cohort of patients with and without dementia diagnosed with general and vascular conditions that required surgery was described. We feel that our review of outcomes for 120 operated patients with a pre-existing diagnosis of dementia compared with the results of an equal number of sex- and treatment-matched controls with several well-balanced clinical variables accurately reflects surgical outcomes among this patient population.

## Conclusions

Patients with pre-existing dementia have a greater than average risk of early death after surgery, and their incidence of fatal complications is higher than that of surgical patients without dementia. The predominant causes of in-hospital mortality after surgery are infectious and cardiac in nature. Patients at greatest risk of early death are those with a higher ASA class, who undergo emergent operations and develop postoperative pulmonary complications. Despite the inferior surgical outcomes and limited life expectancy, the lack of effective alternative therapy may justify a surgical approach for a surgical diagnosis in these difficult-to-treat patients. Further research is needed to develop strategies to optimize the surgical management of patients with dementia in order to address the challenges they present.

## Abbreviations

ARF: Acute renal failure
ASA: The American Society of Anesthesiologists Physical Status classification
BMI: Body mass index
CA: Cardiac arrhythmia
CCI: Comprehensive complication index
CNS: Central nervous system disease
COD: Coexisting disease
COPD: Chronic obstructive lung disease
CRF: Chronic renal failure
DC: Clavien-Dindo classification of complications
GS: General surgery
ICD: International Statistical Classification of Diseases and Related Health Problems
ICU: Intensive care unit
LFRI: Lung failure requiring intubation
LOS: Length of hospital stay
PD: Postoperative delirium
PERI: Pleural effusion requiring intervention
SSI: Surgical site infection
TIA: Transitory ischemic attack
VS: Vascular surgery
